# Recovery of Ammonium Sulfate Solution by Regeneration of Loaded Clinoptilolite

**DOI:** 10.3390/nano12030525

**Published:** 2022-02-02

**Authors:** Stephan Wasielewski, Eduard Rott, Ralf Minke, Heidrun Steinmetz

**Affiliations:** 1 Chair of Sanitary Engineering and Water Recycling, Institute for Sanitary Engineering, Water Quality and Solid Waste Management (ISWA), University of Stuttgart, Bandtaele 2, 70569 Stuttgart, Germany; eduard.rott@iswa.uni-stuttgart.de (E.R.); ralf.minke@iSWa.uni-stuttgart.de (R.M.); 2 Chair of Resource-Efficient Wastewater Technology, Faculty of Civil Engineering, University of Kaiserslautern, Paul-Ehrlich-Str. 14, 67663 Kaiserslautern, Germany; heidrun.steinmetz@bauing.uni-kl.de

**Keywords:** regenerability, wastewater, sludge water, resource recovery, alkalic regeneration, ammonium recovery, sludge liquor, molecular sieve

## Abstract

The zeolite clinoptilolite (CLI) is known to be a very good ion exchanger, as it consists of a three-dimensional structure formed of AlO_4_^−^ and SiO_4_ tetrahedral, which are connected by a common oxygen atom. The micropores formed by this structure (with free diameters in the range of 0.40 nm and 0.72 nm) are fine enough to allow cations and water molecules to enter and be exchanged. CLI is a suitable, inexpensive, and globally available material for removing ammonium from highly-concentrated wastewater and is proven to be selective in ammonium uptake and regeneration since no effect of the provenance of the ammonium (matrix-free NH_4_Cl solution or sludge water) could be found. However, regeneration of the clinoptilolite is necessary to recover the adsorbed ammonium for further use and restore its capability for ion exchange. Within this work, the method by which clinoptilolite, loaded with ammonium (*q* = 8.1–16.6 mg/g) from different sludge waters and ammonium chloride solution, can be regenerated to yield a stoichiometric ammonium sulfate solution (ASS), that could be used, e.g., as a fertilizer, was investigated. A regeneration solution containing Na_2_SO_4_ (0.25 n(Na_2_SO_4_)/n(NH_4_^+^_ads_)) with a varying NaOH ratio (0–2.14 n(NaOH)/n(NH_4_^+^_ads_)) was tested. To obtain a high ammonium concentration in the eluate, a large mass fraction ω of 284 g/kg of CLI in the regeneration solution was applied. The effects of different ammonium loads, different origins of the ammonium, and residual moisture on the necessary components of the regeneration solution, in which an ASS is obtained within a contact time of 10 min at 22 °C, were studied. A stoichiometric ASS from CLI loaded up to a maximum of 13.5 mg/g was obtained with a mixture of 0.25 n(Na_2_SO_4_)/n(NH_4_^+^_ads_) together with 0.8–1.0 n(NaOH)/n(NH_4_^+^_ads_) for dry CLI, and 0.75 n(NaOH)/n(NH_4_^+^_ads_) for CLI with residual moisture.

## 1. Introduction

In general, wastewater is treated using various biological processes. Since ammonium poses a great risk to the environment, it has to be removed at high energy costs taking up a heavy yield on the WWTP capacity. As a by-product of the anaerobic digestion of the sludge arising from these processes, a water stream high in ammonium concentration (500–1000 mg/L NH_4_-N [1,2]), also known as sludge water (SW), is returned to the main course of the WWTP.

The zeolite clinoptilolite (CLI) has been used in many studies as a suitable material for removing ammonium from highly-concentrated wastewater [3,4,5,6,7]. As it consists of a three-dimensional tetrahedral structure formed of AlO_4_^−^ and SiO_4_, connected by a shared oxygen atom, it is known to be a very good ion exchanger with a high selectivity for monovalent ions such as NH_4_^+^ [8,9]. The micropores formed by this structure (with free diameters in the range of 0.40 nm and 0.72 nm) are fine enough to allow the entry and exchange of cations and water molecules [8,10]. CLI has a porosity of 0.34 [11]. This ability is based on the substitution of SiO_4_ by AlO_4_^−^, leading to a negative charge in the structure, which has to be compensated by exchangeable cations such as Na^+^, K^+^, Ca^2+^, and Mg^2+^ [12]. More information on the adsorption mechanism of clinoptilolite can be found in Margeta et al. [8]. In a previous study with Carpathian clinoptilolite powder (CCP 20), 21.0 meq/100 g Na^+^, 49.3 meq/100 g K^+^, 65.6 meq/100 g Ca^2+^, and 3.3 meq/100 g Mg^2+^ were exchanged with 136.9 meq/100 g NH_4_^+^ [13]. Furthermore, CLI is inexpensive and available in various deposits worldwide [14].

Regeneration of the sorbent is necessary to remove the adsorbed ammonium for further use and hereby restore the capability for ion exchange.

The contact of loaded zeolite with sodium-containing regeneration solutions, such as sodium hydroxide and sodium chloride solutions, converts the zeolite into a homionic form, i.e., only one type of cation is adsorbed in the framework structure. From previously published studies, it is reported that the homionic form of CLI has a higher uptake capacity [15,16,17,18], can take up ammonium faster [19,20], and has altered pore diameters (either larger [18] or smaller [19]) and a larger specific surface area [18,19]. In addition, experiments were performed in which the CLI was transformed into a homionic form by directly converting the adsorbed ammonium to elemental nitrogen using NaClO [15]. When subsequently comparing the sorption kinetics and isothermal sorption of both regenerated CLI and homionic CLI, no differences between the properties of CLI were found.

However, Demir et al. [21] published partly contradictory observations. When comparing natural CLI and CLI regenerated with NaCl solution (pH 12.3), they found that regenerated CLI achieved higher loading more rapidly at concentrations of 40–80 mg/L NH_4_^+^. However, at concentrations of 10–20 mg/L NH_4_^+^, they observed that both CLI achieved similarly high loading within the first 5 min to 10 min and, depending on the initial concentration, were in equilibrium with the solution after 30 min of contact time. The authors concluded that the pores and the surface of the CLI were covered with a monolayer of ammonium ions.

In conclusion, it can be deduced from the studies that CLI will be converted to the homionic sodium form as a result of regeneration with sodium-containing regeneration solution and, therefore, will have an equal or even higher uptake capacity as well as higher sorption rates.

Furthermore, the regeneration efficiency can be increased by the addition of NaOH. Guo et al. [22] enhanced the regeneration efficiency from less than 70% to 90–95% by the addition of NaOH (0.032–0.1 M) to a NaCl regeneration solution (58.5 g/L NaCl). However, the maximum regeneration efficiency was achieved with 20 g/L NaCl (0.34 M) and 0.1 M NaOH. Thus, NaOH and the resulting high pH in the regeneration solution improve the regeneration efficiency.

Since most studies did not conduct their experiments with real wastewater matrices, it is unknown to what extent the composition of the CLI is changed by the regeneration or if undesirable compounds are dissolved out of the framework structure of the zeolite. In addition, it is uncertain which parameters are required in the regeneration process to obtain an equimolar ammonium sulfate solution (ASS) that could be used, e.g., as a fertilizer.

The objective of this study was to develop a deeper understanding of the factors influencing the regeneration of ammonium loaded powdered natural clinoptilolite with real wastewater compared to synthetic solutions. Additionally, the objective was to obtain an ASS that could potentially be used as a fertilizer.

## 2. Materials and Methods

### 2.1. Zeolite Samples

Preliminarily studied Slovakian CLI CCP 20 (CCP = Carpathian clinoptilolite powder) showed favorable sorption properties [13] and is suitable to remove ammonium from highly concentrated sludge water [2]. It was obtained from the supplier Labradorit GmbH (Berlin, Germany) in ground and sieved form (particle size smaller than 20 µm) and used for this study.

Prior to the experiments, the CLI was loaded with ammonium either from two different sludge waters (SW) or from an ammonium chloride solution. The SW originated from the mechanical dewatering of anaerobically treated sludge of two different wastewater treatment plants [2]. During normal operation, this sludge water is returned to the main treatment process.

SW_1_ originated from a WWTP treating mainly municipal wastewater using the activated sludge process with upstream denitrification. Iron chloride sulphate is employed for phosphate precipitation. Primary sludge from mechanical treatment, secondary sludge from the biological stage, and precipitated sludge from phosphorus elimination are admixed and anaerobically digested. Before dewatering, the digested sludge is pre-thickened and subsequently dewatered by means of a chamber filter press. For the investigations, the sludge water was taken from the outlet of the chamber filter press.

SW_2_ originated from a WWTP with a high industrial wastewater share. The wastewater is treated by trickling filters and downstream denitrification. A mixture of aluminum and iron salts is employed as a precipitant for phosphate elimination. Sludge from primary and secondary treatment as well as from phosphate elimination are pre-thickened and subsequently anaerobically digested. Afterwards, the digested sludge is dewatered with a chamber filter press, the outlet of which was sampled to gain SW_2_ for the investigations. Of SW_2_, several samples taken at different times were examined.

Matrix-free ammonium chloride solution (*c*_0_ = 1000 mg/L NH_4_-N) was prepared by dissolving NH_4_Cl in distilled water.

The CLI was loaded by contacting it with the respective sludge water, SW_1_ or SW_2_, or NH_4_Cl solution for 30 min under turbulent stirring. Afterwards, the loaded CLI was separated and dried at 105 °C for 24 h before use.

The natural CCP 20 mainly consisted of Si (35.5% (*wt/wt*)), Al (5.4%), K (2.0%), Ca (1.6%), Fe (1.0%), Na (0.4%), Mg (0.3%), Ti (0.1%), Ba (0.08%), and Pb (0.001%), whereas Cr, Ni, As, Rb, Cd, Cs, Ba, Hg, and Tl were below the limit of detection.

### 2.2. Experimental Design

The objective of the experiments was to determine the required composition of the regeneration solution at which the eluate corresponds to a stoichiometric ammonium sulfate solution (ASS). In a stoichiometric ASS, the charge of the monovalent positively charged ammonium cations is balanced by the charge of the divalent negatively charged sulfate anions (molar ratio *r* = 2 n(NH_4_^+^)/n(SO_4_^2−^)).

In preparation of the regeneration solution, varying quantities of NaOH were added to a Na_2_SO_4_ solution since the regeneration efficiency is improved by NaOH concentration or high pH (12.8) [22]. As a result of the high pH value of the regeneration solution (pH_R_ ≈ 12.6–12.9), unquantifiable stripping losses of uncharged NH_3_ were anticipated. The positive charge of ammonium is balanced by the negative charge of OH^−^ ions in the alkaline range. The water-soluble ammonium ion is converted into uncharged ammonia, the water-solubility of which is highly dependent on temperature, among other factors. In turbulent mixing, air entrainment, and elevated temperature, the water solubility of ammonia is low, causing it to strip out. Therefore, the low molar ratio of the regenerate Na_2_SO_4_ was set to *c_n,S_* = 0.25 n(Na_2_SO_4_)/n(NH_4_^+^_ads_). With this ratio, even in the event of an incomplete elution or loss due to stripping effects of ammonium, sufficient sulfate anions were still in solution.

Loaded sorbent (*q* = 8.1–16.6 mg/g) with ammonium from NH_4_Cl, SW_1_, and SW_2_, respectively, was dried (105 °C, 24 h) and 2 g of each was weighed into a centrifuge tube (50 mL). To the sorbent, 3 mL of Na_2_SO_4_ solution (*c_n,S_* = 0.25 n(Na_2_SO_4_)/n(NH_4_^+^_ads_)) and between 0 mL and 2 mL of high-molarity (1 M and 2 M, respectively) NaOH solution (0–2.14 n(NaOH)/n(NH_4_^+^_ads_)) were added, and the missing volume was filled up to 5 mL with double-distilled water, ensuring a mass fraction *ω* of the suspension of 284 g/kg. Because CLI can absorb water to a large extent due to its high porosity, the mass fraction of CLI in the regeneration solution is reported in this work rather than the dry residue. The mass fraction *ω* (g/kg) was calculated according to Equation (1), where *m_CLI_* (g) is the mass of dry loaded CLI and *m_R_* (g) the mass of regeneration solution.

(1) $$\omega =\frac{m_{CLI}}{m_{CLI}{+m}_{R}}\;\cdot \;1000\;g/\mathrm{kg}$$

The suspension was mixed for 10 min in a rotator (uniROTATOR2, LLG Labware, Meckenheim, Germany) at 80 rpm and 22 °C. After subsequent centrifugation at 4000 rpm for 10 min, the supernatant was decanted, collected, and analyzed. From the linear regression of the molar ratio of ammonium and sulfate in the eluate and the NaOH used, it was possible to determine the composition of the regeneration solution necessary to obtain a stoichiometric ASS. The experiments were performed as triplicates, with the error bars reflecting the standard deviation of the arithmetic mean.

In addition, elemental analysis was applied to investigate whether there was a change in the composition of the CCP 20 before regeneration, i.e., in the pre-loaded state and after regeneration.

In order to remove any additional particles or substances retained by the CLI in addition to the ammonium, a cleaning step, e.g., with water, can be provided after the sorption phase. In the course of this, water remains in the interstices of the zeolite particles and pores (residual moisture). To check the influence of the residual moisture on the elution, loaded CCP 20 was brought into contact with water and mixed for 30 min, centrifuged for 10 min at 4000 rpm, and then subjected to regeneration as described above.

### 2.3. Analytical Methods and Chemicals

Ammonium was measured according to German standard DIN 38406-5 [23]. At a pH of about 12.6, ammonium cations and ammonia contained in the sample react with hypochlorite ions and salicylate ions in the presence of sodium pentacyanonitrosylferrate (2-) (nitroprusside sodium) as a catalyst to form a blue dye. The required hypochlorite ions are formed in the alkaline medium by hydrolysis of the dichloroisocyanuric acid ions. The spectral absorbance of the blue dye at 655 nm wavelength is linearly proportional to the ammonium concentration.

For the determination of pH, probes (SenTix 950 + Multi 3430, WTW, Weilheim, Germany) were used.

For determination of the sulfate concentration, the sample (4 mL, pre-diluted for high concentrations) was placed in a plastic cuvette (layer thickness 1 cm) and acidified with 50 µL concentrated nitric acid (HNO_3_). Subsequently, a spatula tip of BaCl_2_ (~200 mg) was added and after 5 min, the absorbance was determined at 450 nm against water (UV/Vis spectrophotometer JASCO V-550, JASCO Corp., Tokio, Japan). By converting the absorbance against a known calibration curve, the sulfate concentration can be calculated. In order to minimize and estimate measurement errors, a standard with a known concentration was analyzed at the same dilution as the samples.

To determine the chemical elements of the zeolite, 0.3 to 0.5 g of the CLI were weighed and mixed with 6 mL HNO_3_ (65%), 4 mL HF (48%), and 2 mL HCl (32%). The mixture was digested by microwave with a selected program run of 10 min at 383 K, then 5 min at 413 K, and finally 9 min at 463 K. Together with the cooling phase, the digestion lasted 64 min.

Heavy metals were analyzed by inductively coupled plasma mass spectrometry (Nexion 2000, Perkin Elmer, Waltham, MA, USA).

NH_4_Cl (p.a.), NaOH (p.a.), and HCl (32%, p.a.) were obtained from VWR International (Radnor, PA, USA). BaCl_2_ (p.a.), Na_2_SO_4_ (p.a.), and HNO_3_ (65%, p.a.) were obtained from Merck KGaA, Darmstadt, Germany.

## 3. Results and Discussion

### 3.1. Effect of Ammonium Pre-Load

Whenever high loadings are achieved, ammonium penetrates deeply into the lattice structure of the zeolite, and therefore more effort is needed to regenerate the zeolite. Thus, the objective of this experiment was to determine if the load of the zeolite has an effect on the ratio of sodium hydroxide in the regenerate and adsorbed ammonium ions (NaOH ratio) required to achieve a stoichiometric ammonium sulfate eluate.

**Figure 1:**
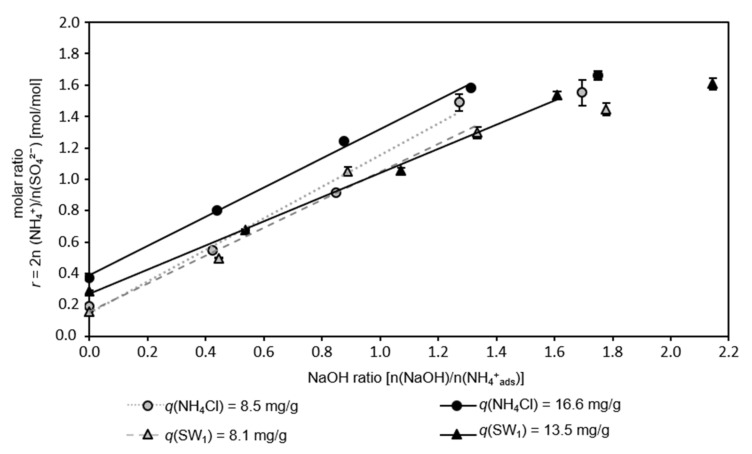
Molar ratio of ammonium cations and sulfate anions in the eluate after regeneration of differently preloaded CCP 20 as a function of NaOH ratio; the linear range of the molar ratio *r* and NaOH ratio is characterized by a linear regression (ω = 284 g/kg; *T* = 22 °C; *t* = 10 min; pH_R_ 12.6–12.9; *c_n,S_* = 0.25 n(Na_2_SO_4_)/n(NH_4_^+^_ads_)).

Figure 1 shows the molar ratio of ammonium and sulfate in the eluate, which occurred after the regeneration of differently loaded sorbents with an increasing NaOH ratio.

Without the addition of sodium hydroxide (NaOH ratio = 0) to the regeneration solution, the molar ratio *r* in the eluate was 0.2–0.4, i.e., there was an excess of sulfate anions. In the case of the sorbents with low load (*q* = 8.1–8.5 mg/g), there was a high excess of sulfate (*r* = 0.2), whereas, in the eluate of the highly loaded sorbents (*q* = 13.5–16.6 mg/g), the excess of sulfate was lower (*r* = 0.3–0.4). A proportionally increasing molar ratio in the eluate (up to *r* = 1.6) was observed with an increasing NaOH ratio. Here, the eluates from the sorbents with loadings *q*(NH_4_Cl) = 8.5 mg/g and *q*(SW_1_) = 8.1 mg/g as well as *q*(SW_1_) = 13.5 mg/g reached nearly identical molar ratios. In contrast, the eluate from the highly loaded CCP 20 (*q*(NH_4_Cl) = 16.6 mg/g) contained observably more ammonium at comparable NaOH input. Apparently, the effectiveness of alkaline regeneration depends on non-ideal loading, i.e., at higher loadings (presumably the near-surface), sorption sites are occupied multiple times [2]. Therefore, the charge of NH_4_^+^ is balanced by OH^−^, thus, in the alkaline range NH_4_^+^ is transformed to uncharged NH_3_, which is no longer retained by the electrostatic forces on or in the framework lattice of the CLI.

A stoichiometric ASS is eluted at a NaOH ratio of 0.8–1.0 n(NaOH)/n(NH_4_^+^_ads_). The coefficients of the regression in the linear range (*r* < 1.6), in which the molar ratio and NaOH ratio were linearly proportional, are shown in Table 1. The coefficients of determination as determined for the differently loaded CCP 20 reflect a high correlation between the NaOH ratio and the molar ratio of ammonium and sulfate ions in the eluate.

Similar results, i.e., improved elution of ammonium by alkalizing the saline regeneration solution, were also reported by Guo et al. [22]. In the study of Guo et al., a notable improvement in regeneration efficiency (from <70% to 90–95%) was reported by increasing the NaOH concentration from 0.032 M to 0.1 M in the regeneration solution.

The regeneration solutions used here had a concentration of 0.114–0.232 M Na^+^ (from Na_2_SO_4_), to which up to 0.8 M NaOH were added, resulting in up to 0.914–1.032 M Na^+^. The Na^+^ concentrations required to elute the stoichiometric ASS (*q*(SW_1_) = 8.1 mg/g: 0.114 M Na^+^ (from Na_2_SO_4_) + 0.182 M NaOH = 0.296 M Na^+^; *q*(SW_1_) = 13.5 mg/g: 0.189 M Na^+^ (from Na_2_SO_4_) + 0.302 M NaOH = 0.491 M Na^+^) were of the same magnitude as Guo et al. [22] had used in their studies (10–50 g/L NaCl + 0.1 M NaOH ≙ 0.271–0.966 M Na^+^). The CCP 20 with a high load from matrix-free NH_4_Cl solution (*q*(NH_4_Cl) = 16.6 mg/g: 0.232 M Na^+^ (from Na_2_SO_4_) + 0.372 M NaOH = 0.604 M Na^+^) also required a correspondingly higher Na^+^ input. The effect of the provenance of ammonium on the regeneration is discussed in Section 3.2.

The ammonium concentration in the eluate, which approximated a stoichiometric ASS, ranged from 1600 mg/L NH_4_-N (from *q*(SW_1_) = 8.1 mg/g) to 2600 mg/L NH_4_-N (from *q*(SW_1_) = 13.5 mg/g) and reached as high as 3900 mg/L NH_4_-N (from *q*(NH_4_Cl) = 16.6 mg/g), depending on the initial load of the CCP 20.

Depending on the flow rate selected, Guo et al. (2013) achieved a concentration maximum between 2000 mg/L and 3000 mg/L NH_4_-N during the regeneration of loaded CLI in columns with a similarly composed regenerant solution. As regeneration progressed, this concentration was strongly diluted by regenerant solution flowing out of the column.

### 3.2. Effect of Residual Moisture

**Figure 2:**
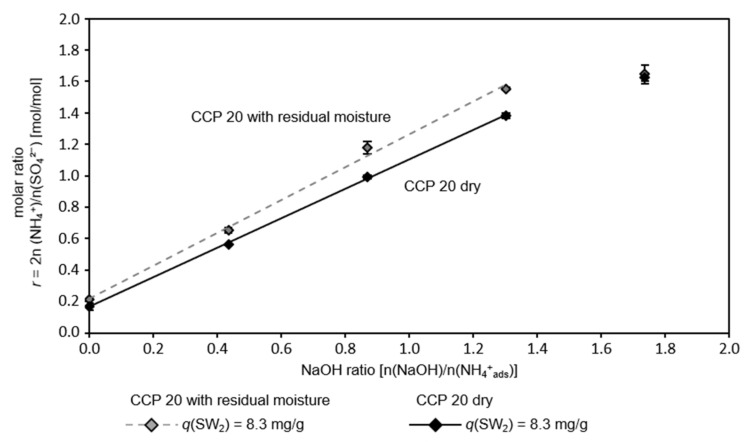
Molar ratio of ammonium cations and sulfate anions in the eluate after regeneration of both, CCP 20 with residual moisture and dry CCP 20, as a function of NaOH ratio; linear regression of molar ratio *r* and NaOH ratio is indicated by lines (*ω* = 284 g/kg; *T* = 22 °C; *t* = 10 min; pH_R_ 12.6–12.9; *c_n,S_* = 0.25 n(Na_2_SO_4_)/n(NH_4_^+^_ads_)).

Since other substances apart from ammonium may adsorb on the CCP 20 from the sludge water, it may be necessary to rinse the loaded zeolite. In this experiment, it should be determined if moisture in the zeolite after washing affects regeneration. Figure 2 depicts the molar ratio *r* in the eluate after the regeneration of loaded CCP 20 with an increasing NaOH ratio. CCP 20 was loaded from SW_2_ and either dried after sorption or washed with water.

Without the addition of NaOH, a ratio of *r* = 0.2 was achieved in the regeneration solution for both the dry CCP 20 and the CCP 20 with residual moisture (excess of sulfate anions in the eluate). With an increasing NaOH ratio of up to 1.3, a proportionally increasing molar ratio in the eluate was observed. The coefficients of the linear regression are listed in Table 2. The NaOH ratio required to obtain a stoichiometric ASS was lower for zeolite with residual moisture (0.75 n(NaOH)/n(NH_4_^+^_ads_)) than for dry zeolite (0.9 n(NaOH)/n(NH_4_^+^_ads_)). Since the pores and interstitial spaces in the zeolite with moisture were already filled with water, the accessibility of deeply stored adsorbed ammonium was higher as compared to dry zeolite.

To elute the stoichiometric ASS, an amount of 0.116 M Na^+^ (from sodium sulfate) and 0.174 M NaOH was required for the zeolite with residual moisture, whereas 0.209 M NaOH was required for the dry zeolite (about 20% more). In total, 1.25 mole Na^+^ per mole adsorbed NH_4_^+^ was required to elute a stoichiometric ASS ((0.25 n(Na_2_SO_4_) + 0.75 n(NaOH))/n(NH_4_^+^_ads_)). The molar ratio of NaOH to Na_2_SO_4_ was approximately 3 = (0.25 n(Na_2_SO_4_) + 0.75 n(NaOH))/n(NH_4_^+^_ads_) = (n(Na_2_SO_4_) + 3 n(NaOH))/4 n(NH_4_^+^_ads_).

Although more ammonium eluted from CCP 20 with residual moisture, the stoichiometric ASS had a lower ammonium concentration (zeolite with residual moisture: *c_Eluate_* ≈ 1200 mg/L NH_4_-N; dry zeolite: *c_Eluate_* ≈ 1600 mg/L NH_4_-N) due to dilution with water contained in the interstitial spaces and pores.

### 3.3. Effect of Ammonium Provenance

**Figure 3:**
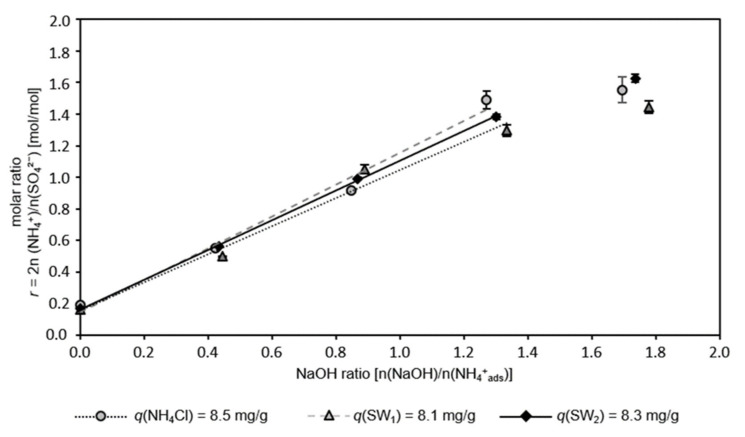
Molar ratio of ammonium cations and sulfate anions in the eluate after regeneration of CCP 20 pre-loaded from different ammonium sources as a function of NaOH ratio; the linear range of the molar ratio *r* and NaOH ratio is characterized by a linear regression (ω = 284 g/kg; *T* = 22 °C; *t* = 10 min; pH_R_ 12.6–12.9; *c_n,S_* = 0.25 n(Na_2_SO_4_)/n(NH_4_^+^_ads_)).

Figure 3 shows the molar ratio of ammonium and sulfate in the eluate occurring from the regeneration of dry sorbents loaded from different ammonium sources (NH_4_Cl solution, SW_1_, and SW_2_) as a function of the NaOH ratio.

By solely employing sodium sulfate solution (NaOH ratio = 0), a molar ratio of *r* = 0.2 (excess of sulfate anions) was reached in the eluate during the regeneration of all three preloaded sorbents. Thus, the achieved molar ratio was independent of the original sample matrix (SW_1_, SW_2_, and NH_4_Cl) from which the ammonium was adsorbed.

With an increasing NaOH ratio of up to 1.3, the corresponding molar ratio in the eluate increased proportionally. Additional ammonium (*r* > 1.3) could be eluted, but the molar ratio in the eluate did not correspond to a stoichiometric ASS. A stoichiometric ASS was eluted with a NaOH ratio of 0.8–1.0 n(NaOH)/n(NH_4_^+^_ads_), i.e., a concentration of 0.114–0.119 M Na^+^ (from sodium sulfate) plus 0.204–0.214 M NaOH (total concentration: 0.318–0.333 M Na^+^) was required in the regeneration solution for the preloaded sorbents studied. The sodium concentration was in the same range as in the previous experiments.

The coefficients of the linear regression of the molar ratio *r* and the NaOH ratio in the linear range (*r* < 1.4) are listed in Table 3. The coefficients of determination as determined for all CCP 20 samples reflect a high correlation of the NaOH ratio and the molar ratio of ammonium and sulfate ions in the eluate.

No influence of sludge water on alkaline regeneration was observed. Apparently, CCP 20 takes up ammonium very selectively [8,9] and releases it during regeneration. Furthermore, this indicates that during the recovery of ammonium from sludge water by means of ion exchange with CLI, undesirable substances from sludge water probably elute only to an extremely small extent into the eluate. These new insights—to our best knowledge, the recovery of ammonium from highly contaminated wastewater by CLI has not been adequately reported—are of great importance for technological set-up and upscaling

### 3.4. Effect of Regeneration on the Composition of CLI

Since the sludge water contains different ions, including heavy metals [2], it is important to determine to what extent they are adsorbed by the CLI and, if applicable, they are later desorbed and present in the eluate after regeneration. Furthermore, cations, e.g., sodium, are adsorbed, and ammonium is released as a result of regeneration. This can be balanced by changes in the composition of the CLI.

**Figure 4:**
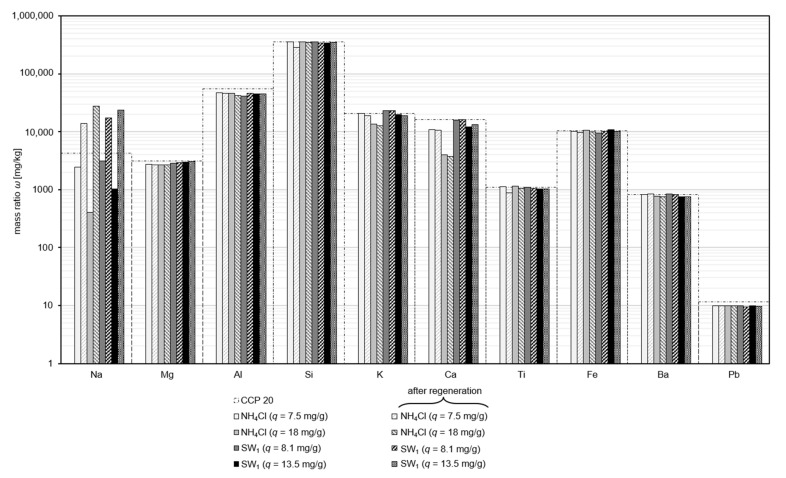
Quantitative elemental analysis of loaded (gray scale) and regenerated (pattern fill) CCP 20 in the case of stoichiometric elution; the composition of natural CCP 20 is plotted as a dashed border (ω = 284 g/kg; *T* = 22 °C; *t* = 10 min; pH_R_ 12.6–12.9; *c_n,S_* = 0.25 n(Na_2_SO_4_)/n(NH_4_^+^_ads_)).

To investigate the composition of CCP 20 before and after regeneration, it was regenerated (ω = 284 g/kg; *T* = 22 °C; *t* = 10 min; pH_R_ 12.6–12.9; *c_n,S_* = 0.25 n(Na_2_SO_4_)/n(NH_4_^+^_ads_); 0.95 n(NaOH)/n(NH_4_^+^_ads_)) and subsequently analyzed for its composition. The quantitative elemental analysis of natural CCP 20, as well as CCP 20 before and after regeneration, are shown in Figure 4.

A noticeable change in composition was present for the element sodium after the sorption process. In the natural CCP 20, the Na content was 4140 mg/kg. As a result of the sorption of ammonium, the Na content of CCP 20 decreased regardless of the sample matrix from 3155 mg/kg (*q*(SW_1_) = 8.1 mg/g) to as low as 400 mg/kg (*q*(NH_4_Cl) = 18 mg/g). This indicates that predominantly Na^+^ ions were exchanged by NH_4_^+^ ions in the adsorption process. Since SW_1_ already contained Na^+^ ions [2], fewer Na^+^ ions were exchanged from CCP 20 than in the case of the matrix-free solution during the sorption process. However, after the regeneration process, the sodium content increased manifold, suggesting that predominantly ammonium was exchanged for sodium.

Regenerated CCP 20 had a much higher Na content (14,000–27,800 mg/kg) as compared to after loading and its natural state. This Na surplus came from Na of the regeneration solution that had exchanged sorption sites of CCP 20 with ammonium.

Furthermore, the Al content of CCP 20 decreased slightly from 46,800 mg/kg to as low as 41,000 mg/kg as a result of the sorption process—presumably, aluminum which is not part of the framework lattice was leached, and therefore its loss has no influence on the ion exchange capacity. The Al content was not changed by the regeneration, so no dealumination is expected due to the alkaline regeneration. It can therefore be assumed that a only small amount of Al was released into the sludge water.

The decrease in Ca content after sorption of ammonium from the NH_4_Cl solution is striking. It is possible that calcium was dissolved out of the framework lattice during the sorption process into the NH_4_Cl solution due to the low pH (arbitrary pH 5.3), or it was also exchanged for ammonium. The pH in the sludge water ranged from 7.9 to 8.0, thus did not leach calcium from the CLI.

Sprynskyy et al. [24] reported that during sorption, with increasing loading, sodium ions were exchanged first before calcium ions. The decrease in Mg content was negligible. Other elements were neither dissolved during the adsorption process nor eluted to any considerable extent.

In Table 4, the exchangeable cations contained in CCP 20 are balanced before and after regeneration.

CCP 20 loaded with NH_4_Cl (*q* = 7.5 mg/g and *q* = 18 mg/g, respectively) exchanged 0.50 meq/g and 1.19 meq/g Na^+^ for 0.30 meq/g and 0.82 meq/g NH_4_^+^ (corresponding to 4.3 mg/g and 11.7 mg/g NH_4_-N, respectively). In the case of CCP 20 loaded with SW_1_ (*q* = 8.1 mg/g and *q* = 13.5 mg/g, respectively), 0.61 meq/g and 0.98 meq/g Na^+^ were exchanged for 0.34 meq/g and 0.59 meq/g NH_4_^+^ (equivalent to 4.9 mg/g and 8.4 mg/g NH_4_-N, respectively).

However, not all cations were completely eluted from the CCP 20 by the sorption of ammonium; consequently, further cations were contained in the loaded CCP 20 in addition to ammonium.

The ion exchange did not occur with the same charge, and the ammonium was not completely eluted from the CCP 20. Further cations were not exchanged or were exchanged to a considerable extent (e.g., K^+^) by regeneration. The relatively large balance error (11–22%) can be ascribed to the improved uptake of cations as a result of alkaline regeneration. Thus, the pore diameters might have been widened, leading to ammonium or cations penetrating deeper into the framework and increasing the uptake capacity or cation exchange capacity [25,26]. A greater capacity was reported after using a regeneration solution of NaCl and NaOH [21,22,27].

The measured K^+^ concentration (31.7–77.7 mg/L) in the eluate was much lower than reported (1000–4000 mg/L K^+^ [22]). Low concentrations of Al (0.029–0.105 mg/L), Si (75.6–145 mg/L), Rb (0.126–0.199 mg/L), Ba (0.089–0.293 mg/L), Ni (0.002–0.026 mg/L), Fe (0.002–0.026 mg/L), and Ti (0.0009–19 mg/L) were also detected in the eluate. Nevertheless, the concentrations are much lower than in the sludge water used [2], with the exception of Ba and Rb, both of which were present at about the same level as in the sludge water. This can be ascribed to the preference of CLI over Ba and Rb in the uptake of ammonium [28]. Apparently, no heavy metals are transferred from the zeolite into the regeneration solution at the high pH value.

## 4. Conclusions

A stoichiometric ASS was obtained from sorbent loaded with a maximum of 13.5 mg/g by a mixture of 0.25 n(Na_2_SO_4_)/n(NH_4_^+^_ads_) and 0.9 n(NaOH)/n(NH_4_^+^_ads_) for dry sorbent, or 0.75 n(NaOH)/n(NH_4_^+^_ads_) for sorbent with residual moisture (ω of 284 g/kg, contact time 10 min). The provenance of the ammonium (matrix-free NH_4_Cl solution or SW_1_ or SW_2_) had no influence on the regeneration process.

The elemental composition of CCP 20 changed as a result of sorption and regeneration. Sorption decreased the amount of Na in the natural CCP 20 (natural: 4140 mg/kg; *q*(SW_1_) = 8.1 mg/g: 3155 mg/kg; *q*(NH_4_Cl) = 18 mg/g: 400 mg/kg). In the regenerated CCP 20, the Na content was much higher (14,000–27,800 mg/kg) since Na was taken up from the regeneration solution. The ammonium was exchanged for sodium; therefore, a sufficiently high concentration must be present in the regeneration solution. In addition, Ca was dissolved out by the acidic NH_4_Cl solution (pH 5.3). Other elements were released from the CLI during sorption only in minor concentrations.

Dealumination of the framework lattice did not occur during regeneration. Low concentrations of K (31.7–77.7 mg/L), Al (0.029–0.105 mg/L), Si (75.6–145 mg/L), Rb (0.126–0.199 mg/L), Ba (0.089–0.293 mg/L), Ni (0.002–0.026 mg/L), Fe (0.002–0.026 mg/L), and Ti (0.0009–19 mg/L) were detected in the eluate. No contamination of the eluate by heavy metals occurred.

Thus, clinoptilolite has proven to be a suitable material for removing ammonium from sludge water and obtaining an ASS by regeneration with sodium sulfate and sodium hydroxide mixture.

Based on the found conditions, it is of interest for future investigations under which parameters the clinoptilolite can be regenerated and possibly reused. In future investigations, it should be examined how often the CLI can be loaded and regenerated. In the course of this, it should also be examined whether the properties of the CLI change noticeably as a result of periodic loading and regeneration.

## Figures and Tables

**Table 1:** Coefficients of the linear regression between the NaOH ratio and the molar ratio of ammonium and sulfate ions in the eluate of differently preloaded CCP 20.

Load of the sorbent	Slope	Intercept	Coefficient of Determination
*q*	*m(x)*	*b*	*r^2^*
8.5 mg/g (NH_4_Cl)	1.0064	0.1467	0.9853
16.6 mg/g (NH_4_Cl)	0.9283	0.3905	0.9969
8.1 mg/g (SW_1_)	0.8913	0.1580	0.9805
13.5 mg/g (SW_1_)	0.7704	0.2692	0.9971

**Table 2:** Coefficients of the linear regression between the NaOH ratio and the molar ratio of ammonium and sulfate ions in the eluate of both, CCP 20 with residual moisture and dry.

Load of the Sorbent	Slope	Intercept	Coefficient of Determination
*q*	*m(x)*	*b*	*r^2^*
8.3 mg/g (SW_2_), dry	0.9395	0.1649	0.9997
8.3 mg/g (SW_2_), with residual moisture	1.0459	0.2193	0.9962

**Table 3:** Coefficients of the linear regression between the NaOH ratio and the molar ratio of ammonium and sulfate ions in the eluate of CCP 20 preloaded from different ammonium sources.

Load of the Sorbent	Slope	Intercept	Coefficient of Determination
*q*	*m(x)*	*b*	*r^2^*
8.5 mg/g (NH_4_Cl)	1.0064	0.1467	0.9853
8.1 mg/g (SW_1_)	0.8913	0.1580	0.9805
8.3 mg/g (SW_2_)	0.9395	0.1649	0.9997

**Table 4:** Cation balance of CCP 20 before and after regeneration.

Cation	Unit	NH_4_Cl		NH_4_Cl		SW_1_		SW_1_	
		7.5 mg/g		18 mg/g		8.1 mg/g		13.5 mg/g	
		Before	After	Before	After	Before	After	Before	After
Na^+^	meq/g	0.11	0.61	0.02	1.21	0.14	0.75	0.04	1.02
K^+^	meq/g	0.53	0.48	0.35	0.33	0.59	0.59	0.50	0.48
Ca^2+^	meq/g	0.14	0.13	0.05	0.05	0.20	0.20	0.15	0.17
Mg^2+^	meq/g	0.06	0.05	0.06	0.06	0.06	0.06	0.06	0.06
NH_4_^+^	meq/g	0.53	0.23	1.26	0.44	0.57	0.23	0.95	0.36
Sum	meq/g	1.36	1.51	1.73	2.08	1.55	1.84	1.71	2.08
Change	%		11		21		19		22
